# Synthesis of OMS Materials and Investigation of Their Acceptor–Donor Characteristics

**DOI:** 10.1007/s10337-012-2284-7

**Published:** 2012-07-31

**Authors:** H. Grajek, J. Paciura-Zadrożna, J. Choma, E. Michalski, Z. Witkiewicz

**Affiliations:** 1 Institute of Chemistry, Military University of Technology, Warsaw, Poland; 2 Institute of Chemistry, Jan Kochanowski University, Kielce, Poland; 3 Institute of Applied Physics, Military University of Technology, Warsaw, Poland; 4 University of Arts and Sciences, Kielce, Poland

**Keywords:** Inverse gas chromatography, Acceptor–donor properties, Mesoporous ordered siliceous adsorbents

## Abstract

Three ordered mesoporous siliceous (OMS) materials known as MCM41s—unmodified MCM-41C16 (“C16”), and two MCM41s with different surface functionalities: MCM-41C16-SH (“C16-SH”) and MCM-41C16-NH_2_ (“C16-NH_2_”)—were synthesized and studied by inverse gas chromatography in order to determine their acceptor–donor properties. The specific retention volumes of nonpolar and polar probes that were chromatographed on these ordered mesoporous silica adsorbents were evaluated under infinite dilution conditions. Two methods were employed to calculate the standard free energy of adsorption, Δ*G*_ads_, of each chromatographed probe on the basis its specific retention volume. These Δ*G*_ads_ values were then employed to estimate the van der Waals contribution and the specific contribution of the free surface energy for each MCM41. DN values (donor numbers, based on the Gutmann scale) and AN* values (acceptor numbers, based on the Riddle–Fowkes scale) were employed to determine the values of parameters that characterize the ability of the MCM41s to act as electron acceptors (parameter: *K*_A_) and donors (parameter: *K*_D_). Considering the different compositions of the probes, each of which has different acceptor–donor properties, a new chromatographic test to supplement the Grob test is suggested.

## Introduction

The family of silica adsorbents known as MCM41s are ordered mesoporous silica (OMS) materials. These adsorbents contain hexagonally ordered mesopores that form a honeycomb structure, although the mesopores are not linked to each other. An important property of these materials is the ability to predict and control the dimensions and volume of the pores in the adsorbent during synthesis. MCM41s have homogeneous cylindrical pores with dimensions ranging from ~1.5 to ~7–8 nm [1]. The pore volume usually ranges from 1.0 to 1.5 cm^3^/g, while the specific surface area ranges from ~900 to ~1,500 m^2^/g. The walls of the mesopores are made of amorphous silica [2].

There are currently very few published papers that address the issue of testing the acceptor–donor properties of ordered mesoporous silica materials via inverse gas chromatography. The interactions between the probe and the ordered mesoporous silica adsorbent can be regarded as van der Waals interactions and/or specific ones. Therefore, the adhesion energy must be divided into several terms corresponding to different dipole interactions (fluctuating dipole–induced dipole, induced dipole–permanent dipole, and permanent dipole–permanent dipole) as well as acceptor–donor interactions (including hydrogen bonds as a subset). To a first approximation, the adhesion energy, *W*_adh_, can be simply expressed as the sum of two terms, *W*_adh_^wdW^, the van der Waals interaction per unit surface area, and *W*_adh_^wdW^, the “specific” interactions (i.e., the same of all interactions that are not van der Waals interactions):

1 $$ W_{\text{adh}} = W_{\text{adh}}^{\text{vdW}} + W_{\text{adh}}^{\text{SP}} . $$

Generally, if there are several sources of specific interactions, then the total molar free energy of adsorption, Δ*G*_ads_, for the adsorption of a probe on the tested MCM41 (which is used to fill the column) can be generally expressed as

2 $$ \Updelta G_{\text{ads}} = \sum\limits_{i} {\left( {\Updelta G_{\text{ads}}^{\text{vdW}} + \Updelta G_{\text{ads}}^{\text{SP}} } \right)} = - RT\ln V_{g(T)}^{(1g)} + {\text{const}}. $$

Here, the value of the constant strongly depends on the chosen reference state of the adsorbed molecule [3]:

3a $$ \Updelta G_{\text{ads}} ( {\text{I)}} = - {\text{RT}}\ln \left( {\frac{{p_{\text{s,g}} }}{{\pi_{\text{s}} S_{\text{BET}} }}V_{g(T)}^{(1g)} } \right), $$

3b $$ \Updelta G_{\text{ads}} ( {\text{II)}} = \Updelta H_{\text{ads}} - T\Updelta S_{\text{ads}} , $$

where $\sum\limits_{i} {\Updelta G_{\text{ads}}^{\text{vdW}} }$ includes all of the van der Waals components of the free energy of adsorption;

$\sum\nolimits_{i} {\Updelta G_{\text{ads}}^{\text{SP}} }$ is the specific component of the free energy of adsorption, which includes all possible sources of interaction other than the van der Waals ones [actually, it is the vertical distance between the total free energy of the polar probe and the total free energy of a hypothetical *n*-alkane on the reference line that has the same value on the abscissa in the plot of Δ*G*_ads_ = *f*_1_(*P*_D_) and $\Updelta G_{\text{ads}} = f_{2} \left( {\alpha_{0} \sqrt {h\upsilon } } \right)$, i.e., the chosen reference states];

$$ V_{g(T)}^{(1g)} = \frac{3}{2}\frac{{\left( {\frac{{p_{\text{i}} }}{{p_{\text{o}} }}} \right)^{2} - 1}}{{\left( {\frac{{p_{\text{i}} }}{{p_{\text{o}} }}} \right)^{3} - 1}}F_{\text{o}} \left( {t_{\text{R}}^{\text{cg}} - t_{\text{m}} } \right)\frac{{T_{\text{c}} }}{{T_{\text{f}} }}\left( {\frac{{p_{\text{o}} - p_{{{\text{H}}_{ 2} {\text{O}}}} }}{{p_{\text{o}} }}} \right)\frac{1}{{m_{\text{ads}} }} $$

is the specific retention volume calculated via the retention times, *t*_R_^cg^, determined for infinite dilution of the probe and with reference to 1 g of the MCM41 in the column [4];

4 $$ t_{\text{R}}^{\text{cg}} = m_{1} = \frac{{\int\limits_{{t_{\text{b}} }}^{{t_{\text{e}} }} {tc(t)dt} }}{{\int\limits_{{t_{\text{b}} }}^{{t_{\text{e}} }} {c(t)dt} }} $$

is the parameter which represents the exact retention time of the center of gravity of the elution peak, corresponding to infinitesimally low coverage of the surface of the MCM41 with probe molecules, and estimated on the basis of the first statistical moment *m*_1_, i.e., the centres of gravity of the peaks [4]; and

*c*(*t*) is the ordinate of the function describing the change in the probe concentration over time (i.e., the dependent variable). In our tests, the raw peaks (the smallest that could be detected), were described by the Weibull equation [5]:

5 $$ \left\{ \begin{gathered} c\left( t \right) = A\left( {\frac{{w_{2} - 1}}{{w_{2} }}} \right)^{{\frac{{1 - w_{ 2} }}{{w_{ 2} }}}} \left[ {\frac{{t - t_{\text{R}}^{\text{cg}} }}{{w_{1} }} + \left( {\frac{{w_{2} - 1}}{{w_{2} }}} \right)^{{\frac{1}{{w_{2} }}}} } \right]^{{w_{2} - 1}} { \exp }\left\{ { - \left[ {\frac{{t - t_{\text{R}}^{\text{cg}} }}{{w_{1} }} + \left( {\frac{{w_{2} - 1}}{{w_{2} }}} \right)^{{\frac{1}{{w_{2} }}}} } \right]^{{w_{2} }} + \frac{{w_{2} - 1}}{{w_{2} }}} \right\}\quad for\quad t > t_{\text{R}}^{\text{cg}} - \left( {\frac{{w_{2} - 1}}{{w_{2} }}} \right)^{{\frac{1}{{w_{2} }}}} \hfill \\ c\left( t \right) = 0\quad for\quad t \le t_{\text{R}}^{\text{cg}} - \left( {\frac{{w_{2} - 1}}{{w_{2} }}} \right)^{{\frac{1}{{w_{2} }}}} \hfill \\ \end{gathered} \right., $$

where

*T*: is the acquisition time for adsorbate elution; i.e., the abscissa of the function (the independent variable).
*t*_m_: is the hold-up time.
*F*_o_: is the carrier gas flow rate, measured by a soap flowmeter at the column temperature.
*T*_c_: is the column temperature.
*T*_f_: is the temperature of the flowmeter.
*p*_i_: is the pressure at the inlet of the column.
*p*_o_: is the ambient pressure.
$$ p_{{{\text{H}}_{ 2} {\text{O}}}} $$
is the pressure of water vapor at the ambient temperature.
*m*_ads_: is the total mass of siliceous material in the chromatographic column.
*p*_s,g_: is the reference pressure, 1 atm (1,01,325 N/m^2^).
π_s_: is the two-dimensional pressure for the adsorbed state, 0.338 × 10^−3^ N/m.
*A*: is the amplitude of the elution peak.
*w*_1_ and *w*_2_: are parameters that are mainly related to the width of the peak. It is important to emphasize that the *w* values relate to the width at different points on the elution peak for different functions [5].
Δ*H*_ads_ and Δ*S*_ads_: are the molar differential enthalpy and entropy of adsorption, calculated on the basis of the equation

6 $$ \ln \frac{{V_{g(T)}^{(1g)} }}{T} = \frac{{ - \Updelta H_{\text{ads}} }}{\text{RT}} + \frac{{\Updelta S_{\text{ads}} }}{R} + \ln \left( {RS_{\text{BET}} m_{\text{ads}} } \right), $$

where

$$ R $$
is the universal gas constant, and
$$ S_{\text{BET}} $$
is the specific surface area of the MCM41 tested, calculated using the BET method.

## Theoretical

Pearson proposed the hard–soft acid–base (HSAB) principle for the generalized Lewis acid–base interaction in order to classify acids and bases based on the absolute hardness [6], whereas Lee summarized Pearson’s proposal in the following way:

i. A hard acid contains an acceptor atom with a high positive charge and a relatively small size. It does not have easily excitable outer electrons, and it is not polarizable [7].
ii. A soft acid contains an acceptor atom with a low positive charge and a relatively large size. It has several easily excitable outer electrons, and it is polarizable [7].
iii. A hard base contains a donor atom of low polarizability that is hard to reduce. It is associated with empty high-energy orbitals, which are therefore inaccessible [7].
iv. A soft base contains a donor atom of high polarizability and low electronegativity. It is easily oxidized, and is associated with unoccupied low-lying orbitals [7].

Isaacs proposed the application of the HSAB principle to organic reactions and use of the frontier orbital approach to investigate electrophilic and nucleophilic interactions [8], and Lee summarized Isaacs’ proposal in a similar manner to his summary of Pearson’s proposal [7]:

i. A hard electrophile (or acid) has a high-energy lowest unoccupied molecular orbital (LUMO), and usually has a positive charge [7]
ii. A soft electrophile has a low-energy LUMO, but it does not necessarily have a positive charge [7]
iii. A hard nucleophile (or base) has a low-energy highest occupied molecular orbital (HOMO), and it usually has a negative charge [7]
iv. A soft nucleophile has a high-energy HOMO, but it does not necessarily possess a negative charge [7]

As is commonly known, the acid–base properties of a solid have a decisive influence on the value of the specific contribution of the enthalpy of adsorption of the solid:

7 $$ - \Updelta H_{\text{ads}}^{\text{SP}} = K_{\text{A}} \times {\text{DN}} + K_{\text{D}} \times {\text{AN}}, $$

where

Δ*H*_ads_^SP^: is the contribution of the specific enthalpy of adsorption of the probe on the tested surface
*K*_A_ and *K*_D_: are parameters characterizing the ability of the tested surface to behave as an electron acceptor or donor, respectively
AN and DN: are the acceptor number and the donor number of the probe, respectively

Liquids can also be characterized by donor (DN) and acceptor (AN) numbers according to the Gutmann acid–base approach [9]:

- The donor number characterizes the basicity or electron-donor ability, which is the molar enthalpy value of the reaction between the base (the electron-donor D) and a reference acceptor, antimony pentachloride (SbCl_5_), in a dilute solution of 1,2-dichloroethane [10]
- The acceptor number characterizes the acidity or electron-acceptor ability, which is defined based on the NMR chemical shift of ^31^P in triethylphosphine, (C_2_H_5_)_3_PO, when it is dissolved in the acceptor solvent A [11].

In chromatographic studies, it is necessary to choose either a strong donor (base) or a strong acceptor (acid), or a species with both of these characteristics (amphoteric).

The donor number (DN) was defined by Gutmann et al. [9, 10] as the negative of the enthalpy of formation of the adduct produced when the organic compound (i.e., the base in question) reacted with the reference Lewis acid, SbCl_5_:

8 $$ \Updelta H_{{{\text{SbCl}}_{ 5} - {\text{base}}}} = {\text{DN}}_{\text{base}} . $$

Gutmann also introduced the concept of an acceptor number (AN) to supplement the DN and measure the strength of the Lewis acidity or electrophilicity of a liquid [9]. The AN values are determined from the magnitudes of the induced chemical shifts in the ^31^P NMR spectrum of triethylphosphine oxide (Et_3_PO), used as a basic probe. Thus, both the AN and DN values are scaled semiempirically, and a given acid–base interaction can be expressed as [12].

9a $$ - \Updelta H = {\text{AN}} \times {\text{DN}}, $$

which is analogous to the *E* and *C* equation [13]

9b $$ - \Updelta H = E_{\text{A}} E_{\text{B}} + C_{\text{A}} C_{\text{B}} , $$

where

Δ*H*: is the enthalpy of adduct formation for the acid–base pair
*E*_A_ and *C*_A_: are empirically determined parameters assigned to each acid
*E*_B_ and *C*_B_: are empirically determined parameters assigned to each base

Generally, the *C* parameters represent the covalent contributions and the *E* values correspond to those arising from the electrostatic interactions between the acidic and the basic components of the adduct.

The main advantage of Gutmann’s donor and acceptor concept is that it recognizes the bifunctionality of substances; however, it is not convenient to distinguish between hard and soft contributions to acid–base behavior.

Due to the incompatibility of the units of the AN and DN numbers and *K*_A_ and *K*_D_, Riddle and Fowkes corrected the AN values for the van der Waals contribution to the chemical shift by determining the *γ*_S_^D^ values from measurements of the surface and interfacial tensions of the test liquids [14]. They determined the enthalpy of formation of the adduct SbCl_5_–(C_2_H_5_)_3_PO and introduced the corrected acceptor number AN*. This enables direct comparison of the *K*_A_ and *K*_D_ values and allows the nature of the surface to be elucidated via the following equation [15]:

10 $$ \frac{{\left( { - \Updelta H_{\text{ads}}^{\text{SP}} } \right)_{i} }}{{{\text{AN}}_{i}^{*} }} = K_{\text{A}} \frac{{{\text{DN}}_{i} }}{{{\text{AN}}_{i}^{*} }} + K_{\text{D}} , $$

where *i* denotes a probe.

Voelkel described another method for determining the *K*_A_ and *K*_D_ parameters that involves the direct use of the Δ*G*_ads_^SP^ value and introduces the contribution of the entropic term [16]:

11a $$ \Updelta G_{\text{ads}}^{\text{SP}} = \Updelta H_{\text{ads}}^{\text{SP}} - T\Updelta S_{\text{ads}}^{\text{SP}} . $$

Thus, the Eq. 11a takes the following form:

11b $$ \frac{{\left( {\Updelta G_{\text{ads}}^{\text{SP}} } \right)_{i} }}{{{\text{AN}}_{i}^{*} }} \cong K_{\text{A}} \frac{{{\text{DN}}_{i} }}{{{\text{AN}}_{i}^{*} }} + K_{\text{D}} , $$

where Δ*G*_ads_^SP^ is the specific component of the free energy. Actually, it is the vertical distance between the total free energy of the polar probe and the total free energy of a hypothetical *n*-alkane on the reference line that has the same value on the abscissa.

The main aim of the work described in this paper was to characterize the acceptor–donor properties of a group of ordered mesoporous functionalized silica adsorbents known as MCM41s. These MCM41s were also manually packed and slurry packed into chromatographic columns [17] and/or anchored to glass capillary columns [18], and investigated by means of inverse gas chromatography.

## Experimental

### Adsorbent Synthesis

Synthesis of the tested MCMs was carried out as follows. Approximately 15.34 g of the surfactant C_16_H_33_(CH_3_)_3_N^+^Br^−^ (*n*-hexadecyltrimethylamino bromide) of purity 99 %, ca. 73 cm^3^ of redistilled water, and ca. 3.6 cm^3^ of 5 M NaOH were placed in a conical flask. The solution obtained was mixed for ca. 1 h, during which time it became milky. After adding surfactant, redistilled water, and NaOH, and tetraethoxysilane [Si(OC_2_H_5_)_4_], the solution became a lyogel, before finally taking on the consistency of porridge after mixing. The resulting adsorbent was denoted “C16.”

The modified adsorbents were synthesized in a similar manner to that described above, but after mixing the surfactant, redistilled water, and NaOH for ca. 1 h, ca. 13.4 cm^3^ of tetraethoxysilane and ca. 0.7 cm^3^ of one of the following modifiers were added:

i. (OC_2_H_5_)_3_Si–C_3_H_6_–SH in the synthesis of MCM41–C16–SH (denoted “C16-SH”)
ii. (OC_2_H_5_)_3_Si–C_3_H_6_–NH_2_ in the synthesis of MCM41–C16–NH_2_ (denoted “C16-NH_2_”)

After adding the reagents, the mixture of tetraethoxysilane and modifier were mixed for ca. 1 h. The consistencies of the solutions changed during mixing: during the syntheses of MCM41C16-SH and MCM41C16-NH_2_, the solutions became gelatinous and then, over time, milky. The next stage in the synthesis of the adsorbents was hydrothermal treatment in an autoclave at 373 K for five days. This treatment was carried out to order the silica structure. The resulting product had a milky consistency. After five days it was washed with ca. 0.7 dm^3^ of redistilled water on Whatman cat. no. 1006 125/6 filter paper and placed in a conical flask. In the conical flask, the product was treated with a mixture of ethanol p.a. and HCl p.a. It was then heated for ca. 6 h at 60 °C, washed with redistilled water, and dried at ca. 105 °C.

The basic characteristics of the MCM41s tested were elucidated on the basis of the results obtained from low-temperature N_2_ adsorption studies, XPS, XRD, and AFM tests, and have already been reported [19].

### Adsorbents and Adsorbates

The characteristics of the C16, C16-SH, and C16-NH_2_ adsorbents tested were elucidated from low-temperature N_2_ adsorption data (reported previously [3]).

The following substances (categorized below according to their interactions with the surfaces of the MCM41s) were employed as probes:

i. Neutral: *n*-alkanes: C_5_–C_8_—0 D and cyclohexane—0.3 D
ii. Acidic: dichloromethane, trichloromethane and tetrachloromethane, which possess* sp* ^3^ hybridization and dipole moments (1.60 D, 1.08 D, and 0 D, respectively), and benzene—0 D
iii. Basic: acetonitrile—3.2 D, tetrahydrofuran—1.75 D, diethyl ether—1.3 D
iv. Amphoteric: acetone—2.9 D, ethyl acetate—1.7 D

As discussed in the “Theoretical” section, the term “acceptor–donor interactions” includes a variety of interactions that researchers have described in different and sometimes ambiguous ways [20]. Therefore, to validate our tests, we first determined the Δ*G*_ads_^SP^ values by plotting the Δ*G*_ads_ values of the *n*-alkanes and the other injected probes as a function of physicochemical parameters (i.e., the molecular descriptors [21] for all of the aforementioned probes)—employing the molar deformation polarization *P*_D_, the deformation polarizability of the molecule *α*_0_, its characteristic frequency ν, Planck’s constant *h*, and $\alpha_{0} \sqrt {h\nu }$ [22] for the abscissa—and subsequently calculated the distances from the experimental points for the probes to the reference line (i.e., the *n*-alkanes line).

### The EDX Tests

Elemental analysis of the surface layers of the MCM41s was performed using a Quantax 200 spectrometer with an EDX XFlash 4010 detector (Bruker-AXS Microanalysis GmbH, Berlin, Germany). The results of this elemental analysis are collated in Table 1.

**Table 1:** The elemental compositions of the MCM41s, determined via EDX tests

MCM41	Elemental composition (%)				
	Si	C	O	S	N
C16	43.03	3.70	53.26	–	–
C16-SH	43.12	3.97	47.65	5.25	–
C16-NH_2_	43.15	3.89	47.83	–	5.12

The total content of impurities was ca. 0.01 %

### Chromatographic Tests

Chromatographic measurements of the aforementioned probes were undertaken using a Unicam (Pay, UK) type 610 gas chromatograph fitted with an on-line Unicam 4880 chromatography data handling system, which was switched on when a sample was injected. The instrument was equipped with a flame ionization detector. The injector and detector temperatures were 396 and 398 K, respectively. Elution peaks of the probes were acquired at 343–393 K and a sampling rate of 10 Hz, employing helium flowing at a rate of 20 ± 0.1 cm^3^/min.

**Fig. 1:**
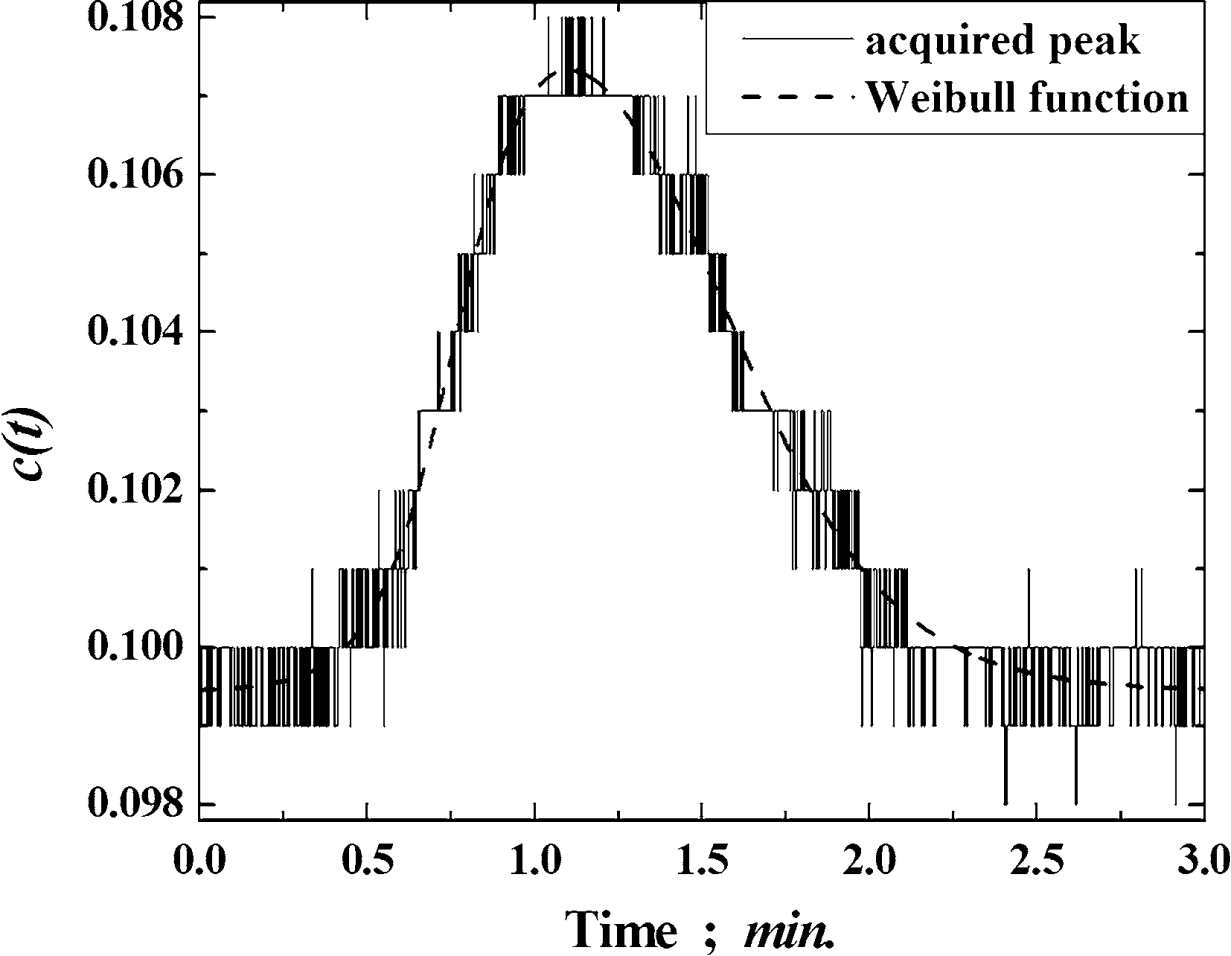
Comparison of the profile of the acquired peak from the chloroform eluted from the C16-NH_2_ adsorbent at 100 °C, with the profile of the Weibull function

**Fig. 2:**
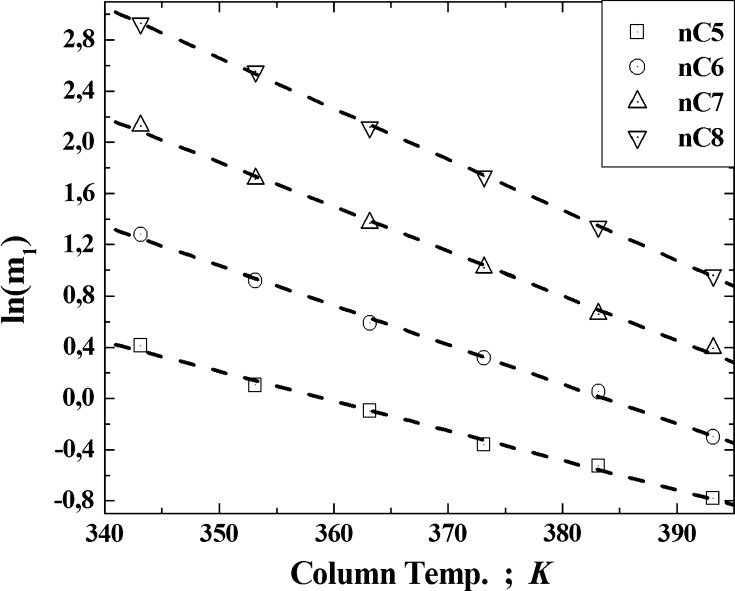
Changes of the values of the logarithms of the average values of the first central statistical moments for *n*-alkanes as the column temperature was increased

Each acquired elution peak was fitted by the Weibull function in order to eliminate small distortions and obtain plausible retention data for the center of gravity of the peak (i.e., the first central statistical moment, *m*_1_ [5]). A comparison of the profile of the peak from the chloroform eluted from the C16-NH_2_ adsorbent at 100 °C and the profile of the Weibull function is depicted in Fig. 1. The changes in the values of the logarithms of the average values of the first central statistical moments for the *n*-alkanes as the column temperature is increased are depicted in Fig. 2, as a way to confirm the correctness of the calculation procedure. For all of the adsorbates tested, the values of the linear regression coefficients were greater than 0.9973. They increased with decreasing column temperature for every adsorbate.

MCM41 samples that had been degassed at 383 K were placed in 65 × 0.4 cm I.D. glass columns each containing an adsorbent bed 10–12 cm in length. The part of the column that was unoccupied by the MCM was filled with glass beads with a mesh size of 80. The filled column was mounted in the chromatograph thermostat and heated at 423 K for 24 h in a helium stream flowing at a rate of 40 cm^3^/min. The amount of probe injected onto the column ranged from 0.05 to 0.005 μl of vapor (for *n*-alkanes, cyclohexane, and benzene) and liquid (for diethyl ether and ethyl acetate).

The important part of these chromatographic tests was the determination of the retention volume of unadsorbed probe: the holdup. The gas holdup time, *t*_m_, can be calculated from the dependence

12 $$ \ln \left[ {\left( {t_{\text{R}i} - t_{\text{m}} } \right)\sqrt {M_{i} } } \right] = a + b \times U\left( z \right), $$

where

*t*_R*i*_: is the retention time of Ar, Kr, Ne, or Xe
*M*_*i*_: is the atomic mass of the aforementioned gas
*U*(*z*): is the Lennard–Jones 10-4 potential function
*a* and *b*: are the coefficients

The justification for this method of calculating the *t*_m_ values has already been presented in detail [4].

Asymmetric elution peaks were obtained for all tested systems, and the retention times of these depended on the amount of probe injected onto the column. The retention times, *t*_R_^cg^, corresponding to infinitesimally low coverage of the MCM41 surface with probe molecules were estimated on the basis of the first statistic moment, *m*_1_ (i.e., the center of gravity of the peak), based on the relation presented in the “Introduction.”

To check whether the raw detector responses changed linearly with probe concentration in the range employed, the function $S_{\text{peak}} F_{\text{o}} = f\left( {m_{\text{a}} q} \right)$ (*S*_peak_ is the peak area, *F*_o_ is the carrier gas flow rate as measured by a soap flowmeter at the column temperature, *m*_a_ is the mass of the probe injected onto the column, and *q* is the chart speed) was employed to produce a calibration plot.

The specific retention volumes were calculated using the retention times determined for infinite dilution of the probe, and for 1 g of the MCM41 in the column, by means of the relation presented in the “Introduction” [4].

## Results and Discussion

When analyzing the chromatographic results obtained for the MCM41s tested, it is necessary to account for the chemical composition of the surface layers. In this context, it is important to emphasize that the surface groups $\equiv {\text{Si}} - \mathop {\text{O}}\limits_{ \bullet \bullet }^{ \bullet \bullet } - {\text{H}}$ and $\equiv {\text{Si}} - \mathop {\text{S}}\limits_{ \bullet \bullet }^{ \bullet \bullet } - {\text{H}}$ are partially acidic owing to *d*-electron cloud vacancies in the silicon atoms (Si^4+^—[Ne]3*s* ^1^3*p* ^3^—is a hard acid according to the hard–soft acid–base principle [6]). Structures containing −SH groups are usually stronger acids than those with −OH groups. The nitrogen atom of an amine can be considered to be* sp* ^3^ hybridized, with the unshared electron pair occupying the orbital 2*s*, $\equiv {\text{Si}} - \mathop {\text{N}}\limits^{ \bullet \bullet } = {\text{H}}_{2}$, and thus acts as an electron donor.

According to Solomons and Fryle [23] the bond dissociation energy of the S–H bond of thiols is about 365 kJ/mol, which is much less than that of the O–H bond of alcohols, ca. 430 kJ/mol. However, −N=H_2_ groups—derivatives of ammonia—are weak bases, as each of these groups can use their unshared electron pair to accept a proton. Therefore, we can expect that the interaction of admolecules with ordered siliceous materials occurs through both nonspecific interactions of *n*-alkanes with the whole adsorbent and specific interactions of polar probes with the surface species.

The elution of probe molecules from the tested column filling as a function of pressure and column temperature is mainly influenced by the size, geometrical structure, and electronic configuration of the probe [4]. Thus, the influence of the electronic structures of admolecules on enthalpies and entropies of adsorption is usually interpreted in terms of dispersive and specific adsorbate–adsorbent and adsorbate–adsorbate interactions [4].

Taking the EDX results into consideration, it is possible to estimate the degree of functionalization of the surface of the MCM41 tested and the electronegativity of the atoms belonging to the outermost layer of the adsorbent surface and the bulk atoms. As is commonly known, an atom’s electronegativity is influenced by both its atomic number and the distance from its valence electrons to the charged nucleus. Thus, the greater its electronegativity, the more an element or probe attracts electrons towards it, which manifests itself in the retention time. The differences in electronegativity between the atoms identified during the EDX tests are collated in Table 2.

**Table 2:** The differences in electronegativity (based on various scales) between the Si and O, S, and N atoms identified during the EDX tests

Electronegativity scale	$\Updelta E_{n} \left( {{\text{O}} - {\text{Si}}} \right)$	$\Updelta E_{n} \left( {{\text{S}} - {\text{Si}}} \right)$	$\Updelta E_{n} \left( {{\text{N}} - {\text{Si}}} \right)$
Pauling scale	~1.54	~0.68	~1.14
Allen scale	~1.694	~0.673	~1.15
Allred–Rochow scale	~1.76	~0.70	~1.33

**Fig. 3:**
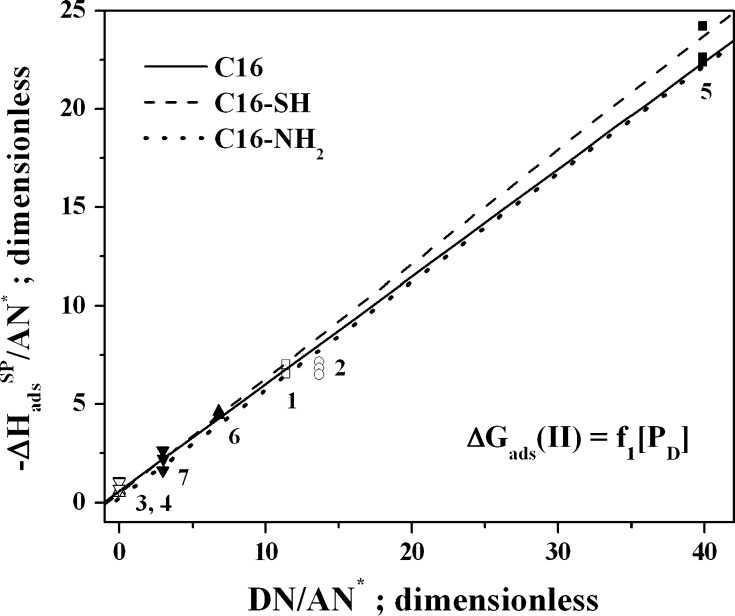
Plots of the $- \Updelta H_{\text{ads}}^{\text{SP}}$ dependencies (Eq. 10) for the MCM41s tested in this work and the following probes: *1* ethyl acetate, *2* diethyl ether, *3* methylene chloride, *4* chloroform, *5* tetrahydrofuran, *6* acetone, *7* acetonitrile

**Fig. 4:**
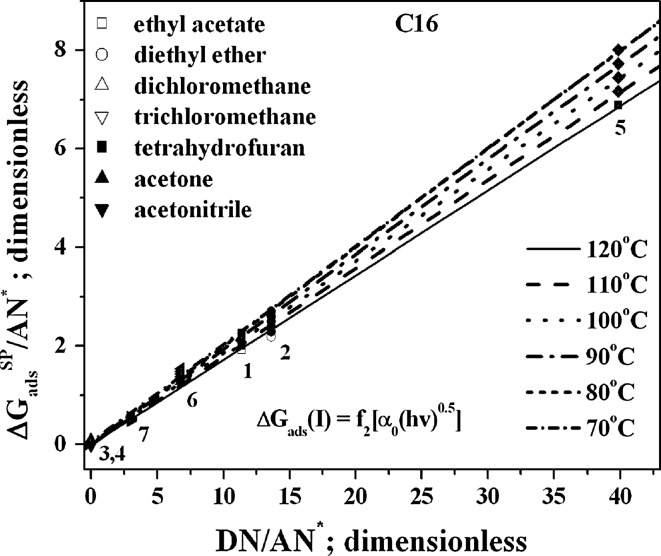
Plots of the $\Updelta G_{\text{ads}}^{\text{SP}}$ dependencies (Eq. 11b) for the C16 material at different column temperatures and the following probes: *1* ethyl acetate, *2* diethyl ether, *3* methylene chloride, *4* chloroform, *5* tetrahydrofuran, *6* acetone, *7* acetonitrile

Based on the theoretical considerations discussed earlier (see the “Introduction” and “Theoretical” sections), the values of the *K*_A_ and *K*_D_ parameters were calculated via Eqs. 10, and 11b. They are collated in Tables 3 and 4, respectively, and the corresponding plots are depicted in Figs. 3 and 4.

**Table 3:** Comparison of the dependencies for Eq. 10 (each dependence is characterized by the value of the linear regression coefficient, *r*, and the standard deviation for linear regression, SD) and the physicochemical parameters plotted on the abscissa without accounting for the entropic effect

Adsorbent	Parameter	∆*G*_ads_ (I)	$\frac{{K_{\text{A}} }}{{K_{\text{D}} }}$	∆*G*_ads_ (II)	$\frac{{K_{\text{A}} }}{{K_{\text{D}} }}$
C16	$P_{\text{D}}$	$\frac{{ - \Updelta H_{\text{ads}}^{\text{SP}} }}{{AN^{*} }} = 0.55 + 0.56\frac{\text{DN}}{{{\text{AN}}^{ *} }}$ *r* = 0.9970, SD = 0.66	1.0	$\frac{{ - \Updelta H_{\text{ads}}^{\text{SP}} }}{{{\text{AN}}^{*} }} = 0.59 + 0.55\frac{\text{DN}}{{{\text{AN}}^{*} }}$ *r* = 0.9974, SD = 0.60	0.9
	$\alpha_{0} \sqrt {h\upsilon }$	$\frac{{ - \Updelta H_{\text{ads}}^{\text{SP}} }}{{{\text{AN}}^{*} }} = 0.42 + 0.56\frac{\text{DN}}{{{\text{AN}}^{*} }}$ *r* = 0.9943, SD = 0.91	1.3	$\frac{{ - \Updelta H_{\text{ads}}^{\text{SP}} }}{{{\text{AN}}^{*} }} = 0.46 + 0.54\frac{\text{DN}}{{{\text{AN}}^{*} }}$ *r* = 0.9948, SD = 0.85	1.2
C16-SH	$P_{\text{D}}$	$\frac{{ - \Updelta H_{\text{ads}}^{\text{SP}} }}{{{\text{AN}}^{*} }} = 0.50 + 0.59\frac{\text{DN}}{{{\text{AN}}^{*} }}$ *r* = 0.9968, SD = 0.72	1.2	$\frac{{ - \Updelta H_{\text{ads}}^{\text{SP}} }}{{{\text{AN}}^{*} }} = 0.48 + 0.58\frac{\text{DN}}{{{\text{AN}}^{*} }}$ *r* = 0.9966, SD = 0.73	0.73
	$\alpha_{0} \sqrt {h\upsilon }$	$\frac{{ - \Updelta H_{\text{ads}}^{\text{SP}} }}{{{\text{AN}}^{*} }} = 0.32 + 0.59\frac{\text{DN}}{{{\text{AN}}^{*} }}$ *r* = 0.9916, SD = 1.17	1.9	$\frac{{ - \Updelta H_{\text{ads}}^{\text{SP}} }}{{{\text{AN}}^{*} }} = 0.29 + 0.58\frac{\text{DN}}{{{\text{AN}}^{*} }}$ *r* = 0.9911 SD = 1.19	2.0
C16-NH_2_	$P_{\text{D}}$	$\frac{{ - \Updelta H_{\text{ads}}^{\text{SP}} }}{{AN^{*} }} = 0.43 + 0.51\frac{\text{DN}}{{{\text{AN}}^{*} }}$ *r* = 0.9974, SD = 0.62	1.2	$\frac{{ - \Updelta H_{\text{ads}}^{\text{SP}} }}{{{\text{AN}}^{*} }} = 0.24 + 0.55\frac{\text{DN}}{{{\text{AN}}^{*} }}$ *r* = 0.9969, SD = 0.73	2.3
	$\alpha_{0} \sqrt {h\upsilon }$	$\frac{{ - \Updelta H_{\text{ads}}^{\text{SP}} }}{{{\text{AN}}^{*} }} = 0.29 + 0.51\frac{\text{DN}}{{{\text{AN}}^{*} }}$ *r* = 0.9964, SD = 0.74	1.8	$\frac{{ - \Updelta H_{\text{ads}}^{\text{SP}} }}{{{\text{AN}}^{*} }} = 0.10 + 0.55\frac{\text{DN}}{{{\text{AN}}^{*} }}$ *r* = 0.9953, SD = 0.90	5.4

**Table 4:** Comparison of the dependencies for Eq. 11b (each dependence is characterized by the value of the linear regression coefficient, *r*, and the standard deviation for linear regression, SD) and the physicochemical parameters plotted on the abscissa, accounting for the entropic effect

Adsorbent	Parameter	*T* (K)	∆*G*_ads_ (I)	$\frac{{K_{\text{A}} }}{{K_{\text{D}} }}$	∆*G*_ads_ (II)	$\frac{{K_{\text{A}} }}{{K_{\text{D}} }}$
C16	$P_{\text{D}}$	120	$\frac{{ - \Updelta G_{\text{ads}}^{\text{SP}} }}{{{\text{AN}}^{*} }} = 0.20 + 0.22\frac{\text{DN}}{{{\text{AN}}^{*} }}$ *r* = 0.9945, SD = 1.60	1.1	$\frac{{ - \Updelta G_{\text{ads}}^{\text{SP}} }}{{{\text{AN}}^{*} }} = 0.20 + 0.22\frac{\text{DN}}{{{\text{AN}}^{*} }}$ *r* = 0.9945, SD = 0.13	1.1
		100	$\frac{{ - \Updelta G_{\text{ads}}^{\text{SP}} }}{{{\text{AN}}^{*} }} = 0.23 + 0.23\frac{\text{DN}}{{{\text{AN}}^{*} }}$ *r* = 0.9949, SD = 1.53	1.0	$\frac{{ - \Updelta G_{\text{ads}}^{\text{SP}} }}{{{\text{AN}}^{*} }} = 0.22 + 0.23\frac{\text{DN}}{{{\text{AN}}^{*} }}$ *r* = 0.9949, SD = 0.16	1.0
	$\alpha_{0} \sqrt {h\upsilon }$	120	$\frac{{ - \Updelta G_{\text{ads}}^{\text{SP}} }}{{{\text{AN}}^{*} }} = 0.15 + 0.22\frac{\text{DN}}{{{\text{AN}}^{*} }}$ *r* = 0.9903, SD = 0.46	1.5	$\frac{{ - \Updelta G_{\text{ads}}^{\text{SP}} }}{{{\text{AN}}^{*} }} = 0.15 + 0.22\frac{\text{DN}}{{{\text{AN}}^{*} }}$ *r* = 0.9903, SD = 0.47	1.5
		100	$\frac{{ - \Updelta G_{\text{ads}}^{\text{SP}} }}{{{\text{AN}}^{*} }} = 0.17 + 0.23\frac{\text{DN}}{{{\text{AN}}^{*} }}$ *r* = 0.9909, SD = 0.48	1.4	$\frac{{ - \Updelta G_{\text{ads}}^{\text{SP}} }}{{{\text{AN}}^{*} }} = 0.16 + 0.23\frac{\text{DN}}{{{\text{AN}}^{*} }}$ *r* = 0.9909, SD = 0.48	1.4
C16-SH	$P_{\text{D}}$	120	$\frac{{ - \Updelta G_{\text{ads}}^{\text{SP}} }}{{{\text{AN}}^{*} }} = 0.16 + 0.21\frac{\text{DN}}{{{\text{AN}}^{*} }}$ *r* = 0.9936, SD = 0.37	1.3	$\frac{{ - \Updelta G_{\text{ads}}^{\text{SP}} }}{{{\text{AN}}^{*} }} = 0.17 + 0.21\frac{\text{DN}}{{{\text{AN}}^{*} }}$ *r* = 0.9933, SD = 0.38	1.3
		100	$\frac{{ - \Updelta G_{\text{ads}}^{\text{SP}} }}{{{\text{AN}}^{*} }} = 0.17 + 0.23\frac{\text{DN}}{{{\text{AN}}^{*} }}$ *r* = 0.9936, SD = 0.41	1.4	$\frac{{ - \Updelta G_{\text{ads}}^{\text{SP}} }}{{{\text{AN}}^{*} }} = 0.19 + 0.23\frac{\text{DN}}{{{\text{AN}}^{*} }}$ *r* = 0.9939, SD = 0.39	1.2
	$\alpha_{0} \sqrt {h\upsilon }$	120	$\frac{{ - \Updelta G_{\text{ads}}^{\text{SP}} }}{{{\text{AN}}^{*} }} = 0.10 + 0.21\frac{\text{DN}}{{{\text{AN}}^{*} }}$ *r* = 0.9883, SD = 2.33	2.1	$\frac{{ - \Updelta G_{\text{ads}}^{\text{SP}} }}{{{\text{AN}}^{*} }} = 0.11 + 0.21\frac{\text{DN}}{{{\text{AN}}^{*} }}$ *r* = 0.9879, SD = 2.36	2.0
		100	$\frac{{ - \Updelta G_{\text{ads}}^{\text{SP}} }}{{{\text{AN}}^{*} }} = 0.10 + 0.23\frac{\text{DN}}{{{\text{AN}}^{*} }}$ *r* = 0.9881, SD = 2.35	2.4	$\frac{{ - \Updelta G_{\text{ads}}^{\text{SP}} }}{{{\text{AN}}^{*} }} = 0.12 + 0.23\frac{\text{DN}}{{{\text{AN}}^{*} }}$ *r* = 0.9885, SD = 2.31	2.0
C16-NH_2_	$P_{\text{D}}$	120	$\frac{{ - \Updelta G_{\text{ads}}^{\text{SP}} }}{{{\text{AN}}^{*} }} = 0.26 + 0.24\frac{\text{DN}}{{{\text{AN}}^{*} }}$ *r* = 0.9942, SD = 0.44	0.9	$\frac{{ - \Updelta G_{\text{ads}}^{\text{SP}} }}{{{\text{AN}}^{*} }} = 0.25 + 0.24\frac{\text{DN}}{{{\text{AN}}^{*} }}$ *r* = 0.9943, SD = 0.43	0.9
		100	$\frac{{ - \Updelta G_{\text{ads}}^{\text{SP}} }}{{{\text{AN}}^{*} }} = 0.26 + 0.25\frac{\text{DN}}{{{\text{AN}}^{*} }}$ *r* = 0.9947, SD = 0.44	1.0	$\frac{{ - \Updelta G_{\text{ads}}^{\text{SP}} }}{{{\text{AN}}^{*} }} = 0.25 + 0.25\frac{\text{DN}}{{{\text{AN}}^{*} }}$ *r* = 0.9947, SD = 0.44	1.0
	$\alpha_{0} \sqrt {h\upsilon }$	120	$\frac{{ - \Updelta G_{\text{ads}}^{\text{SP}} }}{{{\text{AN}}^{*} }} = 0.18 + 0.24\frac{\text{DN}}{{{\text{AN}}^{*} }}$ *r* = 0.9900, SD = 0.57	1.4	$\frac{{ - \Updelta G_{\text{ads}}^{\text{SP}} }}{{{\text{AN}}^{*} }} = 0.17 + 0.24\frac{\text{DN}}{{{\text{AN}}^{*} }}$ *r* = 0.9904, SD = 0.56	1.4
		100	$\frac{{ - \Updelta G_{\text{ads}}^{\text{SP}} }}{{{\text{AN}}^{*} }} = 0.18 + 0.25\frac{\text{DN}}{{{\text{AN}}^{*} }}$ *r* = 0.9911, SD = 0.57	1.4	$\frac{{ - \Updelta G_{\text{ads}}^{\text{SP}} }}{{{\text{AN}}^{*} }} = 0.17 + 0.25\frac{\text{DN}}{{{\text{AN}}^{*} }}$ *r* = 0.9911, SD = 0.57	1.5

The values of *K*_A_ and *K*_D_ reflect the ability of the tested MCM41 surface to act as an electron acceptor and an electron donor, respectively. The acceptor–donor properties of the tested MCMs were characterized on the basis of the recommended values of the quotient $\frac{{K_{\text{A}} }}{{K_{\text{D}} }}$ :

13a $$ \frac{{K_{\text{A}} }}{{K_{\text{D}} }} \ge 1.1,\quad {\text{acidic surface}} $$

13b $$ \frac{{K_{\text{A}} }}{{K_{\text{D}} }} \le 0.9,\quad {\text{basic surface}} $$

13c $$ 0.9 < \frac{{K_{\text{A}} }}{{K_{\text{D}} }} < 1.1\quad {\text{amphoteric surface}} $$

It is important to note that, when both Eqs. 10 and 11b are employed, the $\frac{{K_{\text{A}} }}{{K_{\text{D}} }}$ values are above 0 (see Figs. 3 and 4). It is apparent that the entropic effect has a decisive influence on the chromatographically determined *K*_A_ and *K*_D_ values. The analysis indicates that all of the tested adsorbents have acceptor properties. This seems obvious for C16 and C16-SH because of the acceptor properties of the $\equiv {\text{Si}} - \mathop {\text{O}}\limits_{ \bullet \bullet }^{ \bullet \bullet } {\text{H}}$ and $- {\text{C}}_{ 3} {\text{H}}_{ 6} - \mathop {\text{S}}\limits_{ \bullet \bullet }^{ \bullet \bullet } {\text{H}}$ groups, whereas well-founded doubts arise from the $\frac{{K_{\text{A}} }}{{K_{\text{D}} }}$ values for the C16-NH_2_ adsorbent. These values probably derive from the fact that the relatively high O content has a greater influence on the reported results than the concentration of N atoms (see Tables 1 and 2).

The silanol groups and siloxane bridges have a decisive influence on the acceptor–donor properties of the MCM41 tested. Unfortunately, information on the chromatographic testing of the acceptor–donor properties of siliceous materials is scarce in the literature. Therefore, it is difficult to compare them, because the values of the *K*_A_ and *K*_D_ parameters were calculated on the basis of results obtained using different numbers of probes. It is important to realize that the number of probes taken into account may appear (to researchers) to have only a minor effect on the values of the *K*_A_ and *K*_D_ parameters for different MCM41s. Therefore, we calculated the values of the *K*_A_ and *K*_D_ parameters for all of the polar probes used, and in the second case we omitted acetone. The results obtained are collated in Table 5, and they unambiguously illustrate that the number of probes influences the *K*_A_ and *K*_D_ values.

**Table 5:** The *K*_A_/*K*_D_ values calculated for the adsorbent C16-SH and the physicochemical parameters $P_{\text{D}}$ and $\alpha_{o} \sqrt {h\upsilon },$ (which were plotted on the abscissa, and taking into consideration the entropic effect) when the following probes were used: dichloromethane, trichloromethane, acetonitrile, tetrahydrofuran, diethyl ether, ethyl acetate with acetone, and the same group without acetone

Probes used	Parameter	∆*G*_ads_ (I)					∆*G*_ads_ (II)				
		$K_{\text{A}}$	$K_{\text{D}}$	$\frac{{K_{\text{A}} }}{{K_{\text{D}} }}$	*r*	SD	$K_{\text{A}}$	$K_{\text{D}}$	$\frac{{K_{\text{A}} }}{{K_{\text{D}} }}$	*r*	SD
With acetone	$P_{\text{D}}$	0.59	0.50	1.2	0.9968	0.72	0.58	0.48	0.73	0.9966	0.73
	$\alpha_{o} \sqrt {h\upsilon }$	0.59	0.32	1.9	0.9916	1.17	0.58	0.29	2.0	0.9911	1.19
Without acetone	$P_{\text{D}}$	0.59	0.45	1.3	0.9968	0.81	0.58	0.45	1.3	0.9965	0.83
	$\alpha_{o} \sqrt {h\upsilon }$	0.59	0.20	3.0	0.9920	1.28	0.58	0.19	3.1	0.9913	1.31

Upon analyzing the results collated in Table 5, a critical problem from a chromatographic point of view begins to emerge—there are no sets of probes for these tests—in contrast to, say, the Grob test [24] (the standardized Grob test is a performance test in which variations in peak height or peak area ratio for acidic, basic, and amphoteric probes with respect to an inert solute are measured and compared).

In our tests we injected the molecular probes and polar probes (amphoteric, acidic, and basic) separately, but the Grob test uses a mixture of molecular probes and polar probes, which must be eluted far enough from each other that mutual interactions are avoided as much as possible. Nevertheless, our attempts are not being remanded, in contrast to existing tests that are used to check the quality of chromatographic fillings.

The *n*-alkanes (*n*-decane, *n*-undecane, and *n*-dodecane) are neutral and should elute intact, although partial retention of the alkanes may occur on very inert, nonpolar columns.

The esters (methyl decanoate, methyl undecanoate, and methyl dodecanoate) are amphoteric, so they behave in a similar way to *n*-alkanes.

The alcohols 1-octanol and butane-2,3-diol are hard bases according to the classification of hard and soft acids and bases [25], so in these cases retention is increased by hydrogen bonding. It is well known that chromatographic tests with an alcohol are very informative, because these molecules are more sensitive to adsorption than most other functional groups. The hydroxyl group interactions may be caused by a hydrogen-bonding mechanism.

According to the Grob test, a primary monoalcohol is appropriate for testing a column filled with a phase that has no hydroxyl groups in its structure, such as silicones. Diols are eluted at the start of the chromatogram from columns filled with nonpolar silicone phases. However, carboxylic acids are adsorbed only slightly more strongly than 1-octanol on column fillings without any basic sites, provided that the column filling is not so strongly acidic that it causes tailing of the alcohols.

Acceptor–donor interactions are gauged based on the retention of 2,6-dimethylaniline, 2,6-dimethylphenol, dicyclohexylamine, and 2-ethylhexanoic acid. The Grob test suggests that primary amines form weaker hydrogen bonds than primary alcohols, so they elute faster than primary alcohols from column fillings with acidic sites.

Polar probes with sterically hindered groups are preferred in tests because they allow retention due to hydrogen bonding to be avoided. This usually arises from silanol groups or an oxidized stationary phase, or sometimes sample residues from previous injections.

Dicyclohexylamine and 2-ethylhexanoic acid provide much more stringent tests of acceptor–donor properties than aniline or phenol because their functional groups are so sterically hindered that only acceptor–donor effects are seen in such systems.

*n*-Nonanal is used to assess the capacity of the column to retain saturated aldehydes. This type of adsorption is independent of the amount of hydrogen bonding present.

As presented earlier, the acceptor–donor properties of column fillings represent just one of many sets of information required from chromatographic tests; however, it is necessary to emphasize that they are the most important. Therefore, when employing the Grob test, it is important to realize that acceptor–donor adsorption cannot be interpreted on the basis of well-shaped peaks, because the results are always ambiguous.

In this context, it is necessary to add that London dispersion forces, Keesom dipole–dipole forces, and Debye dipole-induced dipole forces are equally additive, and that hydrogen bonds are only dipole interactions.

Lowering the number of probes employed will have a decisive influence on the results obtained.

The chromatographic test performed using our set of probes will, undoubtedly, characterize the acceptor–donor properties of the column filling, and it should be performed as a complementary test to the Grob test. Since the presence of more than one polar probe exerts a decisive influence on the values of the parameters characterizing the acceptor–donor properties of MCM41s, we believe that our idea should be taken into account when testing the quality of MCM41s as both column fillings and adsorbents anchored to glass capillary columns.

## Conclusions

In the case of probe elution at infinite dilution, the resulting peak usually appears on top of a noisy baseline. Therefore, the description of the peak shape depends greatly on the number of points employed to mathematically model it. The mathematical method employed to describe raw chromatographic peaks allows more plausible retention time data to be obtained for all of the probes used, in an independent and repeatable way.

The IGC method employed here gives more plausible results that take Δ*G*_ads_^SP^ values into account, making it is possible to characterize the acceptor–donor properties of MCM41 surfaces.

The sets of the *K*_A_, *K*_D_, and *K*_A_/*K*_D_ values and the EDX results complement one another very well and enable a deeper explanation of the acceptor–donor properties of the MCM41s when used as column fillings. Good correlation between the EDX results and the IGC ones was obtained for the acceptor–donor properties. This suggests that the specific probe–MCM41 adsorbent interactions are strongly dependent on the elemental compositions of the outermost surface layers and the bulk of the adsorbent.

It is necessary to formulate a set of probes for these tests that will allow unambiguous interpretation of the acceptor–donor properties.

We hope that presenting our opinions about testing the quality of chromatographic fillings as we have done here will aid chromatographists in their attempts to solve other problems.
